# Co-Movement between Carbon Prices and Energy Prices in Time and Frequency Domains: A Wavelet-Based Analysis for Beijing Carbon Emission Trading System

**DOI:** 10.3390/ijerph19095217

**Published:** 2022-04-25

**Authors:** Rundong Luo, Yan Li, Zhicheng Wang, Mengjiao Sun

**Affiliations:** Business School, Shandong University, Weihai 264209, China; luorundong@sdu.edu.cn (R.L.); 201916299@mail.sdu.edu.cn (Z.W.); 202117202@mail.sdu.edu.cn (M.S.)

**Keywords:** Beijing carbon emission trading system, energy prices, wavelet analysis, time–frequency domain

## Abstract

This study aims to investigate the co-movement and lead–lag relationship between carbon prices and energy prices in the time–frequency domain in the carbon emission trading system (ETS) of Beijing. Based on wavelet analysis method, this study examines the weekly data on oil and natural gas prices and carbon prices in Beijing ETS from its establishment in November 2013 to April 2019. Empirical results show the following important findings: (1) Carbon and natural gas prices are mainly negatively correlated, with natural gas prices occupying a leading position in the 12–20 weeks frequency band, indicating that the increase (decrease) of natural gas price will lead to the decrease (increase) of carbon price; (2) carbon and oil prices show an unstable dependence relationship, and their leadership position in the market constantly changes. The partial wavelet coherency and partial phase differences vary greatly in different time–frequency domains, indicating that there is no stable coherency between oil prices and carbon prices. The estimation results prove the existence of coherency between the carbon and energy prices in the Beijing ETS. The research findings of this paper provide quantifiable references for investors to achieve risk control in asset allocation and investment portfolio in the ETS market.

## 1. Introduction

Climate change should be addressed through coordination and consummation of energy policies in the global society [1,2]. The Kyoto Protocol, which was adopted in December 1997, stipulates that by 2008–2012, total emissions of major greenhouse gases in developed countries should have been reduced by 5.2% relative to the baseline in 1990. Since the Kyoto Protocol came into effect, a carbon emission trading system (ETS) has been implemented by a growing number of countries and regions [3]. The global carbon market has emerged since the implementation of the European Union’s ETS in 2005, with both carbon trading volume and turnover increasing rapidly [4].

The rationale for ETS is theoretically straightforward: Operating the carbon market encourages enterprises with low emission-reduction costs to exceed their emission reductions and sell their remaining carbon quotas or greenhouse gas emission reductions to companies with high emission-reduction costs through trading. Such a method helps companies with high emission-reduction costs to achieve the set emission-reduction targets and effectively reduces emission-reduction costs. 

In the carbon market, greenhouse gas emission credits have become a tradable commodity that can be traded between regions and countries [5]. Carbon prices are jointly formed by the supply-and-demand relationship of greenhouse gas emission quotas in the carbon market [6]. The ETS embraces multiple attributes of environment, market, finance, and policy [7]. The price level itself can have varying degrees of effects on improving environmental quality, reducing energy demand, and promoting macroeconomic growth [8].

China has committed to reducing its carbon emissions from peak levels by 2030, and seven pilot carbon ETSs in Beijing, Tianjin, Shanghai, Chongqing, Hubei Province, Guangdong Province, and Shenzhen have been launched [9]. The objective is to compel companies to carry out low-carbon technology innovation through market-oriented means so that low-cost emission reduction can be achieved. The carbon reduction in each pilot has achieved initial results since the pilot initiation of the Chinese ETS. *China’s Policies and Actions for Addressing Climate Change (2019)* states that the country has fulfilled its international commitment to reducing carbon emissions intensity by 40% to 45% by 2020 compared with the levels in 2005. However, China’s carbon market remains troubled by inadequate development and a complex policy environment for energy saving and emission reduction [10]. In this context, improving the operating efficiency of the ETS and using market means to achieve carbon dioxide emission reduction has become a centrally important research question [11].

In this study, carbon and energy prices are dynamically analyzed by using wavelet analysis method based on the data in the Beijing carbon ETS from November 2013 to April 2019. Results reveal the coherency between the carbon and energy prices. The lead–lag relationship between the carbon and energy prices is analyzed through the lag effect of the response between changes in the carbon and energy prices.

The innovation of this study lies in the following aspects: (1) wavelet analysis, which can deal with nonstationarity in economic time series, is adopted for accurate analysis of the structural changes in carbon and energy prices in a nonstationary time series; (2) local coherency analysis is performed from different frequency scales (short and long term), and then the dynamic coherency between carbon and energy prices is studied; and (3) wavelet partial phase difference is used to analyze the lead–lag relationship amongst changes in carbon prices, natural gas prices, and oil prices. 

This study adds quantifiable evidence on the relationship between carbon and energy prices in China’s ETS and contributes to deep understanding of the influencing factors of carbon prices and the interaction between China’s ETS and energy markets. Thus, this study provides a valuable reference for investors in asset allocation and investment portfolio for risk control.

The rest of this paper is organized as follows: Section 2 describes Beijing’s carbon market, Section 3 reviews the literature, Section 4 introduces the methodology and data, Section 5 discusses empirical results, and Section 6 presents the conclusion and recommendations.

## 2. Beijing Carbon Market

The Beijing carbon ETS was established on 28 November 2013. According to data provided by the Beijing Carbon Exchange, by the end of 2018, the total amount of Beijing’s carbon market quota reached 51 million tons, the total exchange volume was 29.07 million tons, the total trading turnover reached CNY 1.049 billion, and the average annual exchange price fluctuated around CNY 50/ton. Among the pilot carbon markets in China, the Beijing carbon market ranks first in volume, turnover, and trading activity. According to the *Beijing Municipal Commission of Development and Reform and the Beijing Environment Exchange (2018)*, the Beijing carbon market covers approximately 1000 companies, including electric power, heat, cement, petrochemicals, other industrial companies, service industries, urban rail transit, and public electric passenger cars. 

The experience of carbon ETS pilots, as pioneers, plays an important role in establishing a national carbon emission market [12,13]. As one of the pilot regions of China’s carbon market, Beijing ETS has achieved good performance and results in reducing emissions. According to the *Annual Report of Beijing Carbon Market 2018*, from 2013 to 2018, the energy consumption per CNY 10,000 of GDP and carbon dioxide emissions in Beijing dropped by 22.5% and 28.2%, respectively, while the city’s energy efficiency ranks first in China. This finding indicates that initial results have been achieved in Beijing through the adoption of the market means to promote energy conservation and emission reduction. In terms of market construction, a relatively complete policy and regulation system of “1 + 1 + N” has formed in the Beijing carbon exchange market. Specifically, the policies include the legislation set forth during the Beijing Municipal People’s Congress on 27 December 2013 (1), local government regulations presented on 28 May 2014 (1), and the specific rules and regulations (N) of the Municipal Commission of Development and Reform and relevant departments in recent years. Meanwhile, a market supervision system based on the maximum position limit and price warning has been formed, which can be an important reference and inspiration for the construction of the national carbon market. The establishment of China’s carbon market is a major impetus to further strengthen the country’s ecological development and promote energy conservation and emission reduction measures.

Beijing ETS was chosen for this study for three main reasons. Firstly, the Beijing carbon market is more active, with trading entities actively trading in the market, its turnover is increasing, and the trading system is operating safely and stably [14]. The price of carbon allowances in Beijing has become an important reference for other pilot cities [15]. Secondly, Beijing carbon market information is disclosed more timely, the market carbon price can reflect market information in a more timely manner, and the construction of the carbon market system is at the forefront of the pilot carbon market [16]. Finally, Beijing’s energy use efficiency is among the highest of all Chinese provinces and cities, indicating that the use of market instruments to promote energy conservation and emission reduction has begun to bear fruit in Beijing [17]. Based on these considerations, this study selects the Beijing carbon market, which has performed well in the carbon market pilots, as the subject of this study.

## 3. Literature Review

A growing body of literature has begun to examine carbon price fluctuations and their influencing factors [18]. The contributing factors of the carbon market can be grouped into internal factors and external ones. The internal factors mainly include the market trading behavior, the adjustment of carbon trading policy, and the uncertainty about economic policy. For example, Wang et al. [19] studied the impact of different types of trading behaviors in the EU ETS on the carbon market. It was found that “compliant transactions” and the inventories of the financial intermediaries have an impact on carbon price trends, while “non-compliant transactions” have an impact on carbon price volatility. Other researchers have confirmed that the EU and China’s carbon trading policies will have an impact on the carbon market [20,21]. Li et al. [22] found that trade policy uncertainty and monetary policy uncertainty will have a positive impact on China’s carbon market prices, while exchange rate policy uncertainty has a negative impact. External factors mainly refer to macroeconomic and weather variables. For example, Bento and Gianfrate [23] proved that macro-level determinants, such as gross domestic product (GDP), the presence of a national carbon price mechanism, and industry attributes, are correlated with the carbon market, and that countries with higher per-capita GDP are usually subject to higher carbon market price levels. Hintermann [24] found that in the EU carbon market, temperature and precipitation affect ETS quota prices in a nonlinear manner. In addition, some studies argue that the performance of financial market, energy market, and foreign exchange market also exert a significant effect on the volatility of carbon prices [25,26,27]. Sun et al. [28] studied the causal relationship between China’s carbon price and the four major energy-intensive stock indexes and found that there is a weak two-way causal relationship between China’s carbon market and the stock market.

Research on the coherency between the carbon market and the energy market has motivated an in-depth study of the influencing factors and effects to form a theoretical underpinning for the development and improvement of the carbon market and its efficiency [29,30,31]. Adekoya [32] found that the prices of energy sources such as crude oil, natural gas, and coal can help investors predict the price of carbon allowances in Europe. Energy prices, such as those of oil and natural gas, are considered amongst the important influencing factors of the carbon price [33]. Zhang and Sun [34] proved that a one-way and time-varying spillover effect exists between the European ETS and the coal and natural gas markets, and no significant spillover effect can be observed between the European carbon market and the crude oil market. However, Ji et al. [35] studied the coherency between the returns and volatility of the carbon energy system under the EU carbon emissions exchange system. Their findings show that Brent crude oil prices play an important role in affecting carbon price fluctuation and risks. Wu, Wang, and Tian [29] proved that the volatility spillover between the coal and carbon emission markets is the strongest by using the recursive graph method and recursive quantitative analysis method. Hammoudeh et al. [36] studied the effect of changes in United States crude oil, natural gas, and other energy prices on carbon prices. Jiang and Chen [37] found that the outbreak of COVID-19 will expand the spillover effect between carbon and energy markets. To sum up, there has been growing evidence of the coherency between the carbon market and the energy market.

For China’s carbon market, few studies have focused on its coherency with the energy markets. Zeng, Nan, Liu, and Chen [15] studied the dynamic relationship between carbon prices in the Beijing carbon market and economic development and energy prices through the structural vector autoregression (SVAR) model. Applying the adaptive lasso method, Guo [38] found that the carbon price in the Shenzhen ETS was mainly affected by the EUR exchange rate and domestic oil prices. Wang et al. [39] simulated the changes in China’s energy system and the trend of carbon emissions by establishing a hybrid energy model to minimize costs. Dong et al. [40] studied the relationship between China’s regional carbon emission intensity and energy structure by using a static spatial econometric model and a panel co-integration model. Wagner et al. [41] investigated the relationship between renewable energy costs and carbon prices in China. Using the vector error correction model, Zhou and Li [42] studied the dynamic relationship amongst energy prices, macroeconomic indicators, air quality, and carbon prices in the Hubei carbon ETS. By using a multi-time series model and an ARCH model, Li and Lei [43] found that the industrial revenue, energy prices, government intervention, and number of participating companies in the carbon ETS in Hubei affected carbon prices considerably. 

Although the above studies shed light on our research, they still have shortcomings. In terms of research perspective, although the coherency between carbon and energy prices in the Chinese carbon market has been investigated, studies are mostly conducted from a static perspective without reflecting the dynamic coherency and leader–follower (lead–lag) relationship between variables in different time and frequency domains. In terms of research method, empirical research on the relationship between carbon and energy prices is mainly conducted by using geographically weighted regression, SVAR, Bayesian SVAR, and other traditional approaches [15,44,45]. These research methods have certain weakness and difficulty in accurately identifying the effective information of carbon prices and energy prices from the perspective of time and frequency domains. Multivariate wavelet analysis can effectively compensate for the inadequacies of traditional methods [46]. Wavelet analysis tools can reflect the changes in variables in the time domain and the short-term and long-term coherency between variables in the frequency domain [47]. Wavelet analysis has been used in research on carbon emissions and carbon assets [48,49,50]. Therefore, a wavelet analysis tool is a powerful alternative to confirm the coherency between carbon prices and energy prices in various time and frequency domains. Based on this, we use the wavelet analysis method to conduct dynamic analysis of carbon price and energy price and explore the correlation between carbon price and energy price. At the same time, the lead–lag relationship between the carbon and energy prices is analyzed through the lag effect of the response between changes in the carbon and energy prices.

## 4. Methods and Data

### 4.1. Research Methods

Wavelet analysis is a mathematical analysis method that was gradually developed in the middle and late 1980s. This method inherits the localized idea of Fourier transformation and overcomes its shortcomings in that the window size is not changed along with frequency [51]. Wavelet theory has become one of the most commonly used tools in the field of signal processing [52]. Its advantages are the following: (1) Analysis is conducted by combining the windows with different frequency fluctuations and different time scales, that is, combining time and frequency domains [53,54]. (2) The method has good localization properties and can find information about structural characteristics hidden in the data that cannot be recognized by other signal methods. (3) Nonstationary time series can be handled and analyzed well. (4)In the long wavelength band, the wavenumber resolution of the overall spectrum of the wavelet transform is higher and the error is lower [55]. On the basis of these excellent characteristics, wavelet analysis is widely used in signal processing, physics, geography, and other fields, and it began to be used in the economic and financial fields in the 1990s [56]. Continuous wavelet transform and wavelet analysis tools, such as wavelet power spectrum, wavelet coherency coefficient, and wavelet phase difference, are introduced in the present paper. In accordance with the research needs of this study, wavelet analysis tools can be extended to cases with three time variables.

#### 4.1.1. Continuous Wavelet Transform

Given a time series *x*(*t*), the expression of its continuous wavelet transform function is

(1) $$W_{x}\left(\tau ,s\right)=\int_{-\infty }^{\infty }x\left(t\right){\bar{\psi }}_{\tau ,s}\left(t\right)dt.$$

In Equation (1), ${\bar{\psi }}_{\tau ,s}\left(t\right)$ indicates a complex conjugation function of the daughter wavelet function $\psi_{\tau ,s}$,

Where $\psi_{\tau ,s}\left(t\right)$ is a sequence of functions obtained after scaling and translation of mother wavelet function ψ(t) [55,57], which can be shown as

(2) $$\psi_{\tau ,s}\left(t\right)=\frac{1}{\sqrt{\left|s\right|}}\psi \left(\frac{t-\tau }{s}\right),s,\tau \in R,s\neq 0.$$

where *s* and τ are the scaling and translation parameters, respectively. When |*s*| > 1, the mother wavelet is stretched and transformed to obtain a high-frequency part with a short duration in the sample time series. When |*s*| < 1, the mother wavelet is compressed and transformed to obtain a low-frequency part with a long duration in the sample time series. The translation transformation is the movement of the mother wavelet function on the time axis. Different values of τ represent different window positions.

The mother wavelet function ψ(t) needs to satisfy the following conditions, which means that the function fluctuates up and down around the time axis:

(3) $$0<\int_{-\infty }^{+\infty }\frac{\psi \left(\omega \right)}{\left|\omega \right|}d\omega <\infty .$$

(4) $$\int_{-\infty }^{+\infty }\psi \left(t\right)dt=0.$$

(5) $$\int_{-\infty }^{+\infty }\psi^{2}\left(t\right)dt=0.$$

The mother wavelet function has numerous forms. In this study, Morlet wavelet is used as the mother wavelet function because it is more commonly used in the analysis of financial and economic time series. The Morlet wavelet is expressed as follows:

(6) $$\psi_{\omega_{0}}\left(t\right)=\pi^{-\frac{1}{4}}e^{i\omega_{0}t}e^{-\frac{t^{2}}{2}}.$$

The value of $\omega_{0}$ deserves special attention. A high value leads to poor localization property of the Morlet wavelet in the time domain, while a small value leads to the poor localization property of the Morlet wavelet in the frequency domain. To effectively balance the effect of these two aspects, $\omega_{0}=6$ is usually considered.

#### 4.1.2. Wavelet Power Spectrum

**Figure 1:**
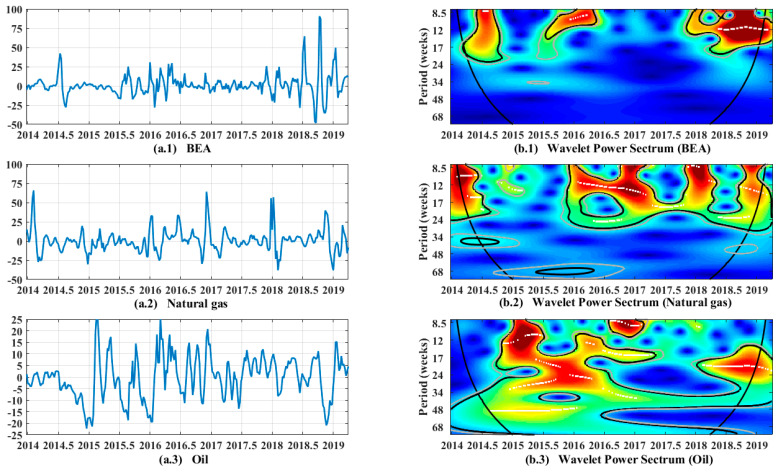
Monthly rate of return and self-wavelet power spectrum. **Note:** (**a.1**–**a.3**) indicates monthly return chart for each time series, and (**b.1**–**b.3**) shows self-wavelet power spectrum.

**Figure 2:**
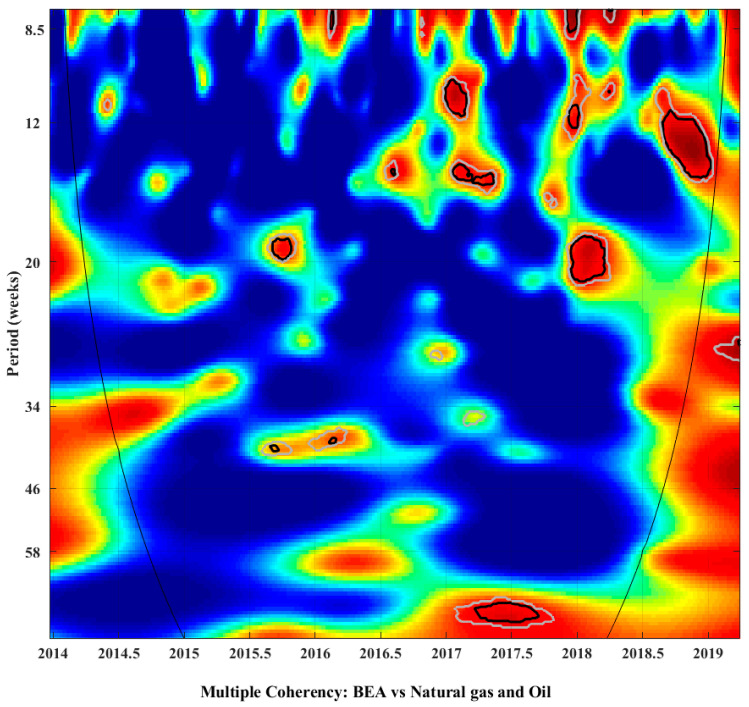
Multiple-wavelet power spectrum between Beijing carbon price and energy (natural gas and oil) prices.

**Figure 3:**
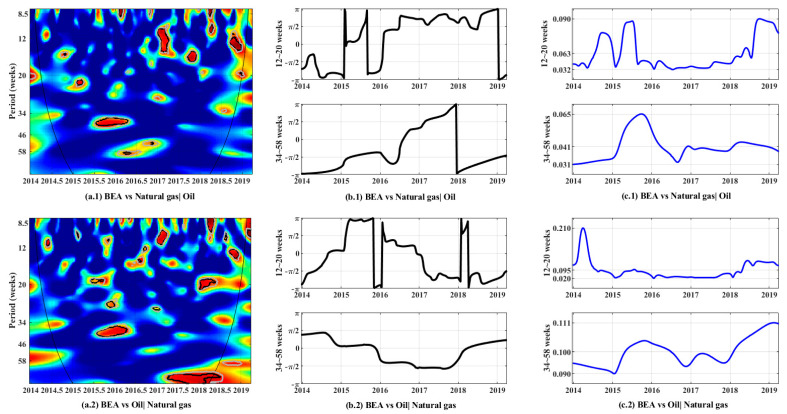
Partial wavelet coherency, phase difference, and wavelet gain. **Note:** (**a**) partial wavelet coherency between Beijing carbon price and natural gas price (**a.1**) or oil price (**a.2**); (**b**) partial wavelet phase difference between Beijing carbon price and natural gas price (**b.1**) or oil price (**b.2**); and (**c**) partial wavelet gain between Beijing carbon price and natural gas price (**c.1**) or oil price (**c.2**).

When the wavelet power spectrum is expressed as the volatility of a single time series under the time and frequency domains, it is called the self-wavelet power spectrum and is defined as ${\left|W_{x}\left(\tau ,s\right)\right|}^{2}$. The volatility in the self-wavelet power spectrum can be indicated by the warm and cool colours in the picture (Figure 1, Figure 2 and Figure 3). The blue part corresponds to the low-power region, indicating that the volatility is low at this time and frequency domain; the red part represents the high-power region, indicating that the volatility is high at this time and frequency domain.

When the wavelet power spectrum is expressed as the coherency of two time series in the time and frequency domains, it is called cross-wavelet power spectrum and defined as $\;{\left|W_{xy}\left(\tau ,s\right)\right|}^{2}={\left|W_{x}\left(\tau ,s\right)\right|}^{2}{\left|\bar{W_{y}}\left(\tau ,s\right)\right|}^{2}$. Similarly, the strength of the coherency between the two time series can be indicated by the warm and cool colours in the cross-wavelet power spectrum. The blue part corresponds to the low-power region, indicating that the correlation of two time series is low at this time and frequency domain. The red part represents the high-power region, indicating that the coherency of two time series is high at this time and frequency domain.

#### 4.1.3. Wavelet Coherency Coefficient and Wavelet Phase Difference

Although the cross-wavelet power spectrum can obtain the correlation between the two time series in the time and frequency domain, knowing which sequence plays a leading role is difficult, that is, the lead–lag relationship between the two time series is unclear. Therefore, the introduction of wavelet coherency coefficients and wavelet phase differences is required.

The wavelet coherency coefficient, which can measure the coherency and size of two time series, is defined as

(7) $$R_{xy}=\frac{\left|s\left(W_{xy}\right)\right|}{{\left[s\left({\left|W_{x}\right|}^{2}\right)s\left({\left|W_{y}\right|}^{2}\right)\right]}^{1/2}}.$$

In Equation (7), s is the smoothing operator achieved by convolution in both time and scale. To further obtain the lead–lag relationship of the time series, we define the wavelet phase difference as

(8) $$\phi_{yx}=tan^{-1}\left(\frac{ℑ\left(W_{xy}\right)}{ℜ\left(W_{xy}\right)}\right).$$

In Equation (8), $ℜ\left(W_{xy}\right)$ and $ℑ\left(W_{xy}\right)$ represent the real and imaginary parts of cross-wavelet power spectrum $W_{xy}$. The different values of wavelet phase difference represent the lead–lag relationship between two time series in specific time and frequency domains. If $\phi_{yx}$ = 0, then *x*(*t*) and *y*(*t*) of the time series are completely positively correlated. If $\phi_{yx}$ = ±π, then the time series are completely negatively correlated. If $\phi_{yx}$ ranges between 0 and π/2, then the series are positively correlated and *y*(*t*) occupies a leading position compared with *x*(*t*). If $\phi_{yx}$ ranges between −π/2 and 0, then the series are negatively correlated and *x*(*t*) occupies a leading position compared with *y*(*t*). If $\phi_{yx}$ ranges between π/2 and π, then the series are negatively correlated and *x*(*t*) occupies a leading position compared with *y*(*t*). If $\phi_{yx}$ ranges between −π and −π/2, then the series are negatively correlated and *y*(*t*) occupies a leading position compared with *x*(*t*).

Wavelet analysis can be extended to cases with multiple time series. This study focuses on three time series. The corresponding wavelet power spectrum is called multiple-wavelet power spectrum, which can be used to analyze the coherency between multiple time series. The warm and cool colors indicate the coherency between the series. Corresponding partial wavelet coherency and partial phase difference indicate research on the coherency and lead–lag relationship between the other two time series, with one time series controlled. Due to the limited space, the paper of Aguiar can be referred to for the specific content of multiple time series [58].

### 4.2. Data

Three variables are considered in the estimation model: carbon exchange price in the Beijing carbon ETS (BEA), natural gas price, and oil price. Carbon dioxide gas emissions originate mainly from the combustion of fossil energy. Different enterprises have different needs for various types of fossil energy as a result of changes in fossil energy prices. When the price of fossil energy is low, a company increasingly uses fossil energy. This condition leads to greater carbon dioxide emissions, which raises the demand for carbon subsidies and further increases carbon prices, and vice versa. Therefore, oil and gas prices are selected as two variables that affect carbon prices. The present study uses weekly data. Carbon price is the transaction price data on the Beijing carbon market from its establishment until 11 April 2019; these data are obtained from the China Carbon Emissions Exchange Network. Due to the high correlation between the Chinese energy market and the international energy market [59] and the lack of relevant data in China, natural gas prices adopt the natural gas spot price of Port Henry, Louisiana, and the oil price adopts the Russian ESPO crude oil spot price. 

## 5. Empirical Results

### 5.1. Empirical Findings of Self-Wavelet Power Spectrum

Self-wavelet power spectrums of carbon price, natural gas price, and oil price in the Beijing carbon market are given in the following. A preliminary analysis on the characteristics of the three time series for carbon price, natural gas price, and oil price from the perspective of volatility shows the actual oscillations of each time series. On the left side of Figure 1, the monthly rate of return of the BEA price and trend chart of the monthly increase rate of natural gas and oil prices are plotted. On the right side of Figure 1, the self-wavelet power spectrum is plotted, with a time span from the end of 2013 to the 14th week of 2019.

In terms of the energy spectrum, the abscissa represents time and the ordinate represents frequency, which is represented by a time period in weeks. Frequency and period can be converted by using the following formula: frequency = 1/period. At each time and frequency, the color of the wavelet power spectrum shows the fluctuation degree of the time series. The color ranges from dark blue to deep red. The deep blue color indicates weaker volatility, while the deep red color indicates stronger volatility. The part inside the red area, that is, the part indicated by the white line, is the time–frequency domain with the most volatility. The black and grey circles represent significance levels of 5% and 10%, respectively. The black conic line indicates the cone of influence, which identifies the wavelet power spectrum region and existence of edge effects. Aguiar’s wavelets toolkit [58] allows current research using wavelet analysis to proceed smoothly. The toolkit can be run with MATLAB, which facilitates the research work of scholars. We thank them for their development and sharing of wavelets tools.

The results of self-wavelet power spectrum are presented in Figure 1, which helps identify the volatility of carbon prices and natural gas and oil prices in the Beijing carbon market under different timescales. Figure 1a.1 shows that the carbon price in the Beijing carbon ETS exhibits a relatively stable fluctuation overall, while strong fluctuations occurred in several areas, which is consistent with the red region in the power spectrum in Figure 1b.1. Days with strong fluctuations are usually concentrated from mid-2014, mid-2015 to mid-2016, and from early 2018 to 2019, which represents the high-frequency domain (less than 17 weeks). In general, the carbon price in the Beijing carbon ETS has been relatively stable. The local fluctuations may be due to the following reasons: in mid-2014, the Beijing carbon ETS conducted its first annual compliance work since its establishment. During this period, emission-controlling companies that have limited knowledge of the carbon market decided to participate so that they could complete the year’s emission-reduction tasks. Furthermore, the secondary market relaxed the conditions for institutional investor participation and introduced the participation of individual investors, making carbon trading active at this stage and thereby leading to large fluctuations in carbon prices. From mid-2015 to mid-2016, the Beijing carbon ETS had more abundant exchange products and initially exerted its central market function, which resulted in considerable activity. In 2018, the Beijing carbon ETS committed to promoting the formation of a multilevel carbon financial market system that coordinates onsite and offsite co-movement, which makes the carbon market active and prices volatile.

The price of natural gas always exhibits obvious cyclical fluctuations, and large fluctuations occur in the fourth quarter of each year. This condition occurs mainly because the increased heating demand, inventory data, and cold air lead to fluctuations in gas prices as the weather gradually cools down [60]. This condition corresponds to the time interval in which the red region appears in the power spectrum, and volatility is higher under the high-frequency domain (periods under 17 weeks), as shown in Figure 1b.2.

In comparison, oil prices are more susceptible to political and economic factors [61]; thus, the oil price is more volatile. Figure 1a.3 shows that the oil price was on a downward trend before 2015, whereas it rapidly increased after 2015. Since then, the price continuously declined and increased at a great amplitude. In terms of oil prices, the more volatile dates are mainly from 2015 to 2017, which are high-frequency domain with a period less than 24 weeks. This condition may have occurred because of the recovery of the world economy in 2015, which effectively promoted the recovery of the global crude oil market. The oil price increased sharply and then fluctuated sharply in the next two years. Oil prices are susceptible to demand and competition, trade prospects, and politics [62], which are also important reasons for oil price fluctuations.

### 5.2. Empirical Findings of Multiple-Wavelet Power Spectrum

Multiple-wavelet power spectrum is helpful in identifying the coherency of multiple time series. Figure 2 shows an estimate of the multiple coherency between the carbon price and the other two time series (natural gas price and oil price), indicating the time and frequency domains when the coherency is the strongest.

Figure 2 shows that from the latter half of 2015 to the end of 2018, the carbon ETS is related to the natural gas and oil markets. In addition, the span of each black circle line is less than half a year, which indicates that no significant long-term coherency exists between the carbon and energy markets. Furthermore, in the high-frequency domain (12–20 weeks), the carbon price and the natural gas and oil price in the Beijing carbon ETS in the two periods of the first half of 2017 and 2018 exerted a significant coherency. This coherency can be reflected in the red area distribution of the multiple power spectrum. In the low-frequency domain (34–58 weeks), two circles exist at the same time point. In general, the relationship between the carbon price and the natural gas and oil price in Beijing’s carbon ETS is hardly affected by long-term and continuous changes, whereas it is more affected by short-term price shocks. The influence on carbon prices and natural gas and oil prices intensifies over time, as proven by the fact that the significantly coherent time–frequency domains in 2017 and 2018 are more concentrated.

Multiple-wavelet coherency presents evidence of coherency between carbon and energy prices (natural gas and oil) at different frequency and time domains, illustrating the importance of the relationship between carbon and energy prices (natural gas and oil). However, the relationship between the two variables cannot be distinguished by this information alone. Therefore, further analysis should be based on the partial wavelet coherency and partial phase difference. 

### 5.3. Empirical Results of Partial Wavelet Coherency and Partial Phase Differences

This study employed partial wavelet coherency and partial phase difference to identify the lead–lag relationship of carbon and natural gas and oil prices in the Beijing carbon ETS as well as evaluate the dynamic relationship between these prices in the time–frequency domain.

Figure 3 describes partial wavelet coherency, partial wavelet phase difference, and partial wavelet gain between carbon price and each energy price (after another energy price is controlled), thereby reflecting the effect of one variable on another. The middle and right sides of Figure 3 are selected to display the wavelet phase difference and wavelet gain of two frequency bands, which are, respectively, 12–20 and 34–58 weeks, to intuitively reflect the coherency information. This finding is consistent with the coherent and statistically significant frequency bands in Figure 2.

In some partial wavelet coherency diagrams, the coherency color ranges from blue (low coherency) to red (high coherency). For wavelet phase difference, the horizontal axis represents the time scale and the vertical axis represents the phase difference, ranging from −π to +π. The research results prove several findings.

Firstly, the partial coherency (Figure 3a.1) and phase difference (Figure 3b.1) results between the carbon and natural gas prices are obtained from the top part of Figure 3. Three important regions with high coherency overlapped with the highly coherent regions estimated in Figure 2. In the low-frequency domain (34–58 weeks) from the second half of 2015 to the first quarter of 2016, the phase difference ranges between −π/2 and 0, indicating that carbon and natural gas prices have a positive coherency. Fluctuations in natural gas prices lead to fluctuations in carbon prices, that is, natural gas is in a leading position. Another two regions are in the high-frequency domain (12–20 weeks), including the first half of 2017 and the second half of 2018. The phase difference ranges between π/2 and π, indicating that carbon and natural gas prices have a negative coherency. An increase in natural gas prices lead to a decrease in carbon prices. The preceding research results indicate the following: (1) In the short term, as in the first half of 2017 and the second half of 2018, the carbon price of the Beijing carbon ETS is negatively related to the price of natural gas, and changes in natural gas prices lead to changes in carbon prices. (2) Over time, the dependence between carbon and natural gas prices intensifies due to the continuous improvement of the carbon price formation mechanism of the Beijing carbon ETS. Partial wavelet gain also shows that, compared with the first half of 2017, the price gains fluctuated more in the second half of 2018, indicating that natural gas and carbon prices are greatly affected by external shocks. Prior to this, the relationship between carbon prices and natural gas prices has also been studied. Duan et al. [63] found that natural gas prices can have an opposite effect on carbon prices through two channels, the aggregated carbon demand effect and the fuel switching mechanism. Hammoudeh, Nguyen, and Sousa [45] also demonstrated that an increase in the price of natural gas reduces the purchase demand of firms and reduces carbon emissions, thus causing the price of carbon to fall. However, Batten et al. [64] show that the effect of natural gas prices on carbon prices is not significant. Our findings confirm the conclusions of the first two studies.

Secondly, the partial coherency (Figure 3a.2) and phase difference (Figure 3b.2) results between the carbon and oil prices are obtained from the bottom part of Figure 3. The regional distribution of this coherency is scattered, indicating that only a short-term co-movement exists between the carbon and oil markets. Therefore, the co-movement relationship between the two is more affected by short-term shocks than by long-term persistence. To further explain the relationship between carbon prices and oil prices, three important regions are selected for further analysis in the high-frequency (period of 12–20 weeks) and low-frequency (period of 34–58 weeks) domains. These three regions have a high coherency at 10% significance level, as shown in Figure 2 and Figure 3. The first region lies in the low-frequency domain from the fourth quarter of 2015 to the first quarter of 2016, whose phase difference ranges between −π/2 and 0. This result indicates that carbon and natural gas prices have a positive coherency. Oil is in a leading position in that fluctuations in oil prices lead to fluctuations in carbon prices. In the first quarter of 2018, the second region lies in the high-frequency domain, where the phase difference ranges between π/2 and π. This result indicates that carbon and natural gas prices have a positive coherency. Oil is in a leading position in that fluctuations in oil prices lead to fluctuations in carbon prices. In the fourth quarter of 2018, the third region lies in the high-frequency domain, where phase difference ranges between −π and −π/2. This finding indicates that carbon and oil prices have a negative coherency. Carbon is in a leading position in that fluctuations in carbon prices lead to fluctuations in oil prices. The analysis results based on the aforementioned three highly coherent regions show that the carbon and oil prices have an unstable dependence relationship, and their leadership position in the market constantly changes. This result is consistent with the complexity of oil price changes and the fact that the carbon price formation mechanism of the Beijing ETS is incomplete. In addition, partial wavelet gain graph shows that partial gain is stable in both frequency bands, and the value is close to 0.1, which is approximately twice the partial gain between carbon and natural gas prices. The relationship between oil and carbon prices has also been explored by previous researchers. Duan, Ren, Shi, Mishra, and Yan [63] found that oil prices can positively affect the price of natural gas through aggregated carbon demand effects. Hammoudeh, Nguyen, and Sousa [45] found that an increase in crude oil price promotes carbon price in the short run but suppresses it in the long run. In the short run, our findings are consistent with previous studies. However, in the longer term, we believe that the dominant relationship between the price of carbon and the price of oil is changing and there is no clear relationship.

## 6. Conclusions and Policy Recommendations

Understanding the influencing factors of carbon price changes is crucial for studying carbon market prices. In this study, empirical analysis of the coherency and lead–lag relationship between carbon prices and energy prices (natural gas and oil) of the Beijing carbon ETS in the time and frequency domains is performed by using wavelet analysis tools, including partial wavelet coherency and partial phase difference. The conclusions help carbon market participants make appropriate investment and portfolio decisions based on the relationship between the carbon and energy markets. Moreover, the conclusions also have important policy implications for market regulators. For example, risk control and management of market exchanges should be strengthened, and reasonable exchange rules, such as price restrictions and risk reserves, should be formulated to ensure the security and stability of market transactions.

The analysis reveals the following: (1) Multiple-wavelet coherency results show that the carbon price and energy price series are in the high-frequency domain around 2017 and from 2018 to early 2019 (less than 20 weeks), indicating a significantly strong coherency. However, the low-frequency domain (more than 34 weeks) shows a significant coherency in the local period from the second half of 2015 to the middle of 2016. Therefore, the coherency between carbon prices and energy price series has strong coherency in the short term and weak coherency in the long term. The estimation results indicate the correlation between the carbon market and the energy market, especially in the short term. (2) Partial wavelet coherency results of carbon and natural gas prices reflect that these prices have a significantly high coherency in the short term (specifically, the first half of 2017 and the second half of 2018). In the high-frequency band, the phase difference ranges between π/2 and π more than half the time, indicating that carbon and natural gas prices have a negative coherency, in that changes in natural gas prices lead to changes in carbon prices. (3) The results of partial coherency and partial phase difference between the carbon price and the oil price series vary greatly in different time–frequency domains. The lead–lag relationship between the two in the high-frequency and low-frequency bands also changes constantly. Therefore, the carbon and oil prices show an unstable dependence relationship. In conclusion, these prices in the Beijing carbon market have a close relationship.

Based on the research results, the following recommendations are presented:

(1) Investors in the carbon market should pay close attention to energy markets that are strongly coherent with the carbon market. Our findings suggest that the relationship between carbon prices in the Beijing carbon market and the natural gas and oil markets is more influenced by short-term shocks than by long-term persistent factors. In the short term, gas prices and carbon market prices are negatively correlated. Moreover, natural gas prices move earlier than carbon market prices. Although oil price movements are positively correlated with carbon market price movements in the short term, the leadership of the two prices is not certain. This suggests that investors should take full account of the sources of carbon price changes and trends in energy market influences when making long- and short-term investment decisions about the carbon market. Investors can not only use price changes in natural gas to predict carbon price changes in the carbon market, but they can also hedge their risk by investing in the natural gas market for risk control purposes.
(2) Companies should adjust their energy consumption structure to achieve the optimal carbon emission reduction strategy based on the coherency and fluctuation mechanism between the carbon and energy markets. Our findings suggest that in the short term, the carbon price in the carbon market is negatively correlated with the price of natural gas and positively correlated with the price of oil. Therefore, companies may consider reducing their carbon emissions by increasing the proportion of natural gas in their energy consumption to compress costs when the carbon price rises.
(3) Regulatory institutions should pay close attention to the possible negative effects of the carbon market bubble. Our findings show that although the trend of carbon price volatility in the Beijing carbon market has been relatively stable overall, strong local fluctuations have emerged. Therefore, regulators should carry out effective risk control by establishing a sound market supervision mechanism so that the carbon emissions trading market can effectively achieve energy saving and emission reduction.
(4) Governments need to ensure price stability and secure supply of energy. Our findings show that energy markets are closely linked to carbon markets, and that stability in energy markets helps to ensure the effective functioning of carbon markets. Therefore, in order to fulfil the role of carbon markets in environmental sustainability, it is necessary to ensure a stable and secure supply of energy and to continue to promote the use of renewable energy.

Future research can be further explored in terms of both the research object and the factors influencing the carbon market. In terms of the research object, the official opening of China’s national carbon emissions trading market was in July 2021. The relationship between the national carbon market price and the energy price can be further explored by taking the national carbon market as the research object. In terms of carbon market influencing factors, researchers could also consider the linkages between other energy markets and the carbon market, such as the renewable energy market. This could provide more reference for government policy-making and investors’ investment decisions.

## Data Availability

Not applicable.
